# Advances of Exosomal miRNAs in Breast Cancer Progression and Diagnosis

**DOI:** 10.3390/diagnostics11112151

**Published:** 2021-11-20

**Authors:** Wenwen Chen, Zhongyu Li, Pengwei Deng, Zhengnan Li, Yuhai Xu, Hongjing Li, Wentao Su, Jianhua Qin

**Affiliations:** 1 CAS Key Laboratory of Separation Science for Analytical Chemistry, Dalian Institute of Chemical Physics, Chinese Academy of Sciences, Dalian 116023, China; chenwenwen@dicp.ac.cn (W.C.); dengpengwei@dicp.ac.cn (P.D.); 2 University of Chinese Academy of Sciences, Beijing 100049, China; 3 College of Life Science, Dalian Minzu University, Dalian 116600, China; lizhongyu2019@yeah.net; 4 Clinical Laboratory, Dalian University Affiliated Xinhua Hospital, Dalian 116021, China; lzn04@163.com; 5 First Affiliated Hospital of Dalian Medical University, Dalian 116000, China; xyh114@icloud.com (Y.X.); lhj68430@163.com (H.L.); 6 School of Food Science and Technology, Dalian Polytechnic University, Dalian 116034, China; 7 Institute for Stem Cell and Regeneration, Chinese Academy of Sciences, Beijing 100049, China; 8 CAS Centre for Excellence in Brain Science and Intelligence Technology, Chinese Academy of Sciences, Shanghai 200031, China

**Keywords:** exosome, miRNAs, breast cancer, diagnosis, potential biomarkers

## Abstract

Breast cancer is one of the most commonly diagnosed malignancies and the leading cause of cancer death in women worldwide. Although many factors associated with breast cancer have been identified, the definite etiology of breast cancer is still unclear. In addition, early diagnosis of breast cancer remains challenging. Exosomes are membrane-bound nanovesicles secreted by most types of cells and contain a series of biologically important molecules, such as lipids, proteins, and miRNAs, etc. Emerging evidence shows that exosomes can affect the status of cells by transmitting substances and messages among cells and are involved in various physiological and pathological processes. In breast cancer, exosomes play a significant role in breast tumorigenesis and progression through transfer miRNAs which can be potential biomarkers for early diagnosis of breast cancer. This review discusses the potential utility of exosomal miRNAs in breast cancer progression such as tumorigenesis, metastasis, immune regulation and drug resistance, and further in breast cancer diagnosis.

## 1. Introduction

Breast cancer has been the most commonly diagnosed cancer worldwide, with an estimated 2.3 million new cases (11.7%) in 2020. It is also one of the leading causes of cancer death with a mortality rate of 6.9% [1,2]. Several genetic and environmental risk factors have been proved to favor breast cancer development [3,4,5,6], however, the exact cause of breast cancer still remains unclear. The five-year survival rates of breast cancer patients are decreased with the malignant degree of the tumor, with more than 95% for localized breast cancer and less than 25% after metastasis [7]. Early diagnosis and the control of tumor procession are of great importance for the mortality of breast cancer patients. Biomedical imaging combined with tissue biopsy remains the most widely used method for detecting breast cancer [8], despite the fact that it can only detect breast cancer with obvious focus. Liquid biopsy including exosomes [9], circulating tumor cells (CTCs) [10], and circulating tumor deoxyribonucleic acids (ctDNAs) [11] has been recently proposed as a promising diagnosis method in oncology because it is less invasive and can be detected in the early stage of breast cancer without obvious focus.

Exosomes are nanoscale extracellular vesicles released by all cells of prokaryotes and eukaryotes [12]. They inherit many constituents from their donor cells, including proteins [13,14], lipids, nucleic acids [15], and metabolites, which play important roles in the transmission of messages and exchange of substances among cells [16]. Emerging evidence shows that exosomes can affect the physiological status of cells and have significant effects on adaptive immunity, inflammatory processes, and tumorigenesis processes through transfer micro-ribonucleic acids (miRNAs) [17,18,19]. MiRNAs are a group of noncoding ribonucleic acid (RNA) with 20–25 nucleotides which always regulate the post-transcriptional level of gene expression negatively [20]. It has been well recognized that miRNAs are involved in the diagnosis, initiation, progression, prognosis, and response to treatment of breast cancer [21,22]. Compared with free ones, the exosomal miRNAs are more stable since the phospholipid bilayer surrounding exosomes can protect them from being degraded by nuclease in the body fluids [23,24].

In this review, we focus on exosomal miRNAs associated with breast cancer. Since substantial evidence predicts that exosomal miRNAs are essential for breast cancer procession and can be used for diagnosis [25,26], we introduce the recent novel findings from those two aspects. For the role of exosomal miRNAs in breast cancer procession, we mainly introduce the aspects of the tumor microenvironment, tumorigenesis, invasion and migration, immune regulation, and drug resistance. For the cancer diagnosis, we mainly introduce the biological sources, summarize the miRNAs which have the potential to be biomarkers for breast cancer diagnosis, and present detection methods reported by previous articles. Finally, we discuss the future opportunities, challenges, and perspectives on how exosomal miRNAs can accelerate their clinical applications in breast cancer.

This systematic review was conducted in PubMed and Web of Science. The publication date was all years (1900–2021). The search identified a total of 271 articles with the keywords “breast cancer” AND “exosome” AND “miRNA”. Then we analyzed these articles. Seventy-eight publications met the inclusion criteria and were included in the systematic review.

## 2. Exosomal miRNAs in Breast Cancer Progression

The tumor microenvironment (TME) often refers to areas close to solid tumors. Despite breast cancer cells, TME also contains a large number of other different types of cells, including cancer-associated fibroblasts (CAFs), vascular endothelial cells (VECs), immune cells, adipocytes, and myoepithelial cells, etc. In addition, some non-cellular components are also involved, including extracellular matrix (ECM), exosomes, signaling molecules, or soluble cytokines [27,28,29,30]. Additionally, TME differs from normal tissues in high interstitial fluid pressure, hypoxia, and acidity, etc.

**Figure 1:**
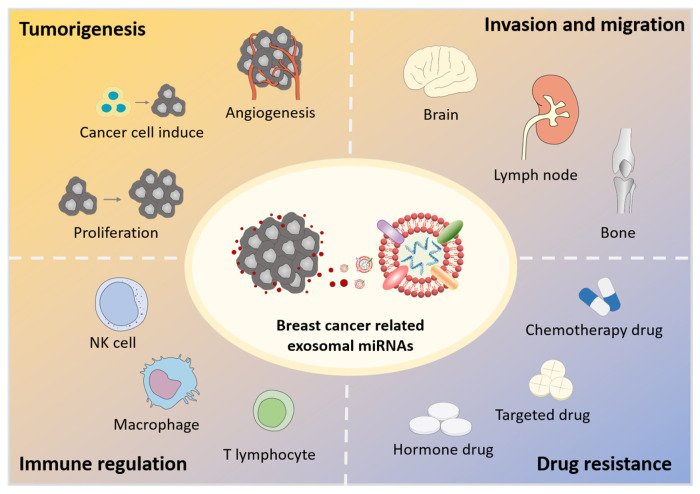
Breast cancer-related exosomal miRNAs in breast cancer progression.

In the tumor microenvironment, cancer-related cells usually exhibit enhanced exosomes release to communicate with adjacent or distant cells. These exosomes mediate the intercellular communications between cancer cells, adjacent stromal cells, mesenchymal stem cells, and immune cells by exchanging bioactive substances such as miRNAs and proteins, resulting in the development of the tumor microenvironment [31,32,33]. This communication network is crucial for almost all major tumor hallmarks, such as tumorigenesis, pre-metastasis niche formation, invasion and migration, immune regulation, and drug resistance (Figure 1). The existing literature shows that exosomes function mainly by transferring miRNAs, which can regulate their target mRNAs post-transcriptionally thus leading to the expression/inhibition of target genes [26]. Therefore, exosomal miRNAs play important roles in the tumorigenesis and progression of breast cancer.

### 2.1. Tumorigenesis

Tumorigenesis is the process of abnormal proliferation of cells formed by the loss of regulation of the normal cells’ growth at the gene level due to various factors. Its biological basis is gene abnormalities. The pathogenic factor is that somatic gene mutation leads to normal gene deletions and disordered gene expressions, which affect the biological and genetic activities of cells and forms tumor cells that are different from normal cells in morphology, metabolism, and function.

Breast cancer cell-derived exosomal miRNAs have been implicated in tumorigenesis [34,35]. Cancer exosomes mediate effective and rapid mRNAs silencing to reprogram the target cell transcriptome through miRNAs. Exosomes with RNA-induced silencing complex-associated (RISC-associated) miRNAs can induce tumor formation by nontumorigenic mouse mammary cells (MCF10A cells) [36]. Breast cancer patients’ exosomes switch nontumorigenic epithelial cells into tumors in a Dicer-dependent manner [36]. Additionally, miRNA-205 affects breast cancer cells proliferation via targeting E2F1 [37].

Exosomal miRNAs also participate in the regulation of cancer-related fibroblast induce and angiogenesis [38]. Baroni et al. [39] found exosomal miR-9 could affect the statuses of human normal breast fibroblasts, enhancing the switch from normal fibroblasts (NFs) to cancer-associated fibroblasts (CAFs), thus promoting tumor growth. miR-9 secreted by CAFs can be transferred to receptor NFs through exosomes and mainly involve extracellular matrix remodeling and cell motility pathways. Jung et al. [40] used a miR-210 specific reporting system to observe miR-210 mediated metastasis from hypoxic breast cancer cells to adjacent cells. Through in vitro and in vivo visualization, they found that miR-210 was transmitted through exosomes in the tumor microenvironment, and it was associated with the regulation of vascular remodeling related genes, including Ephrin A3 and PTP1B, thus, promoting angiogenesis. These results suggest that miRNAs from breast cancer cells spread through exosomes to adjacent cancer cells in the tumor microenvironment and affect tumor tumorigenesis.

### 2.2. Invasion and Migration

Breast cancer is the most common malignancy in women, and 20% of them may develop metastases, which is the main cause of breast cancer death. About 6% of patients with breast cancer have distant metastases in lung/pleura, liver, bone, non-axillary lymph nodes, and brain [41,42,43,44]. In 1889, Stephen Paget put forward the famous “seed and soil” metastasis hypothesis, which believes that tumor cells can form metastasis only in a suitable tissue and organ microenvironment [45]. With the development of technology, the metastasis mechanism of tumors is constantly improved. Nowadays, it has been found that a tumor can actively change the microenvironment of metastasis and contribute to pre-metastatic niche formation by secreting exosomes, while there are few examples of microenvironments providing convenience for tumor cells directly [46]. The key process required for the invasiveness of breast cancer to secondary organs is cancer cell invasion, which can be mediated by determined cell interaction mechanisms such as collective invasion, epithelial-to-mesenchymal transition, and macrophage cancer cell feedback loop. These involve multiple interactions between tumor cells and stromal cell subsets and are carried out through direct intercellular adhesion, soluble factor signal transduction, and extracellular matrix (ECM) reconstruction [47,48].

Yuan et al. [49] used xenograft models to study the function of breast cancer-related exosomes on bone metastasis. They indicated that breast cancer cells promoted bone metastasis via transferring exosomal miR-21 to osteoclasts, which could facilitate osteoclastogenesis through regulating PDCD4 and forming a pre-metastatic niche. While marrow stroma can transfer exosomal miRNAs to breast cancer cells to impair metastases [50]. Wang et al. [51] studied the lymph node metastasis of breast cancer. They identified exosomal miRNAs from plasma of breast cancer patients with/without lymph node metastasis and found miR-363-5p, which was significantly downregulated in lymph node-positive patients, could modulate platelet-derived growth factor (PDGF) signaling activity by targeting PDGFB, thus, inhibiting breast cancer cell proliferation and migration to lymph node. Besides, lymphatic endothelial cells can promote breast cancer metastasis through exosomal miRNAs including miR-503-3p, miR-4269, and miR-30e-3p [52]. Other miRNAs, such as miR-7641 [53], miR-155 which targets PTEN and DUSP14 [54], and miR-1226-3p which targets aquaporin-5 [55] have also been reported to associate with breast cancer invasiveness and migration.

Breast cancer stem cells are a subtype of cancer cells with stem-like characteristics. Their development is closely related to the successful metastasis cascade of cancer cells. Cancer-associated fibroblast exosomes with low miR-7641 can promote the stemness of breast cancer cells through HIF-1 alpha [53]. Exosomal miR-130a-3p has been reported to inhibit migration and invasion by regulating RAB5B in human breast cancer stem-like cells [56]. In addition, tumor-associated macrophages can also promote the invasion of breast cancer through the exosomes secreted by macrophages, which can transfer carcinogenic miRNAs into breast cancer cells [57,58].

### 2.3. Immune Regulation

TME also contains a large number of immune cells, including lymphocytes, dendritic cells, monocytes/macrophages, granulocytes and hypertrophic cells, which involve or relate to immune responses. In breast cancer, exosomal miRNAs also participate in the communication between cancer cells and immune cells, thus, regulating adaptive immunity [59]. Breast cancer cells can escape the detection of the immune system through exosome-mediated secretions of proinflammatory cytokines from macrophages and decreases in the cytotoxicity of NK and T-cells.

Tumor-associated macrophages (TAMs) play a critical role in the tumor inflammatory microenvironment. Guo et al. [60] reported that mouse 4T1 breast cancer cell-derived exosomes enhanced IL-1β, IL-6, and TNF α expressions of TAMs. This is mainly because miR-183-5p, which inhibits the expression of PPP2CA, can be transferred from breast cancer cells to macrophages through exosomes, thus, promoting the secretion of proinflammatory cytokines and contributing to tumor progression in breast cancer.

Immune escape of breast cancer cells is important in the pathogenesis of breast cancer. Endoplasmic reticulum (ER) stress can be produced by destroying protein homeostasis. MiRNA mediated mRNA translation inhibition has been widely studied in regulating ER stress and immune escape in human cancer. Yao et al. [61] reported that in breast cancer, exosomal miR-27a-3p increased PD-L1 expression via MAGI2/PTEN/PI3K axis, thus, promoting immune evasion. Jiang et al. [62] found that both miR-181a and miR-9 could promote the expansion and infiltration of immature early myeloid-derived suppressor cells (eMDSCs), and have a strong inhibitory effect on T cell immunity in humans and mice by targeting SOCS3 and PIAS3 respectively. This may provide a potential therapeutic target for the treatment of IL-6 (high) breast cancer.

### 2.4. Drug Resistance

The current death rate of breast cancer has decreased due to improved early monitoring and advanced treatment strategies. Treatment strategies for breast cancer usually combine surgeries with a variety of adjuvant treatments, such as radiotherapy, chemotherapy, targeted therapy, hormone therapy, or a combination thereof. Nevertheless, resistance to therapeutic drugs remains a big obstacle to the success of systematic treatments [63]. The drug resistance of breast cancer cells arises from different mechanisms, among which the drug resistance mediated by exosomal miRNAs has attracted much attention. Emerging evidence reveals that the up-regulation/down-regulation of miRNAs can induce the drug resistance of breast cancer cells through various signal pathways [7,64,65].

Doxorubicin (Adriamycin) [66,67], docetaxel [68], paclitaxel [69], and cisplatin [70] are the commonly used chemotherapeutic drugs in breast cancer therapies. They inhibit the process of breast cancer either by killing tumor cells or arresting tumor cells divisions [71]. Several miRNAs from docetaxel-resistant cells derived exosomes have been proved can modulate target genes associated with mTOR, TGF-beta, MAPK, PI3K/Akt, and Wnt signaling pathways, thus, participating in kinase activities interfering, transcription regulation, protein binding, and protein phosphorylation [72,73]. For example, exosomal miR-100, miR-222, and miR-30a were implicated in breast cancer cell’s resistance to adriamycin and docetaxel [67]. Some modulators of estrogen receptor-α, such as tamoxifen, a commonly used hormone therapy drug, have also been studied [74]. Exosomal miR-9-5p augments the resistance of breast cancer cells to tamoxifen by down-regulating ADIPOQ [75]. Exosomal miRNAs involved in mediating therapeutic drugs resistance may provide a new target for therapeutic intervention.

Drug resistant breast cells can affect the properties of normal cells through exosomal miRNAs. Ozawa et al. [76] isolated extracellular vesicles from triple-negative breast cancer cells and used these vesicles to treat non-tumorigenic breast cells. They found vesicles from cancer cells could promote the proliferation and drug resistance of normal cells by changes in miRNAs associated with cell proliferation, invasion, and apoptosis. This indicates drug-resistant breast cancer cells can change gene expression in sensitive cells by transferring specific miRNAs through exosomes, so as to manipulate a more deleterious microenvironment and transmit drug resistance.

In addition, some upstream factors which affect miRNAs have also been reported. For example, it has been found that β-elemene can regulate the expression of multidrug resistance specific miRNAs in cells, thereby affecting the content of exosomes, reducing the drug resistance through exosomes, and reversing the drug resistance of breast cancer cells [77]. D Rhamnose β-hederin, which could decrease the formation and release of exosomes and reduce the expressions of the most abundant miRNAs (miR-16, miR-23a, miR-24, miR-26a, and miR-27a) in docetaxel-resistant related exosomes, has been used to reverse the chemoresistance of breast cancer cells by regulating the resistance transmission mediated by exosomes [78]. Exosomal miRNAs may be considered as excellent biomarkers for the determination of specific drug resistance in breast cancer therapy and regulating miRNAs in exosomes may help us reduce the resistance of breast cancer cells.

Finally, we summarized miRNAs involved in breast cancer progression and showed them in Table 1.

## 3. Exosomal miRNAs in Breast Cancer Diagnosis

Breast cancer is a diverse disease with different subtypes and stages [84]. Traditional diagnostic methods, such as mammography [85] and tissue biopsy [86], are very effective but they are limited by the need for the smallest tumor size and may lead to radiation exposure. In addition, not all breast tumors can be found by mammography in the early stage. The specificity of mammography is 62.7% with a sensitivity ranging from 62.2 to 89.5% [87]. Based on these facts, multiple researchers have paid attention to blood-based biomarkers, which can help detect breast cancer in infancy before it spreads from the primary site. miRNAs show great potentials in this regard [87]. As mentioned above, these short and non-encoding RNA sequences are involved in the tumorigenesis and progression of breast cancer [21,88]. However, the lack of standardized methods makes it difficult to implement in a clinical environment. Whole blood [89], plasma [90], and serum [91] all have been reported as sources of breast cancer-related miRNAs. Using miR-10b as an example, it has been observed a significant upregulation in the serum [91] has no significant difference on the whole blood [92] of breast cancer patients when compared with healthy individuals. In addition, researchers are working to discover miRNAs that may distinguish breast cancer subtypes from each other.

Compared to free miRNAs in whole blood or serum, miRNAs in exosomes are more stable and reliable since the phospholipid bilayer surrounding exosomes can protect them from being degraded by nuclease in the body fluids. Therefore, exosomal miRNAs have been a promising biomarker for breast cancer diagnosis and attached more and more attention.

### 3.1. Sources for Isolating Breast Cancer Related Exosomal miRNAs

Almost all body fluids contain exosomes, such as blood, urine, milk, sweat, various tissue fluids, and even tear [12,93]. Exosomes separated from several biological samples have been extensively studied to isolate breast cancer-related exosomal miRNAs, including serum [94], plasma [95], and tear [96].

The serum is the main source for the study of breast cancer-related exosomal miRNAs. It is reported that the exosomes in breast cancer patients’ serum contain RNA-induced silencing-loading complex proteins, TRBP, Dicer, and AGO2, which can process pre-miRNAs into mature miRNAs. And the level of exosomes in the serum of breast cancer patients is higher than that of healthy donors [36]. From the diagnostic point of view, miRNAs in circulating exosomes can reflect the composition of donor breast cancer cells and the response of the tumor microenvironment to the growth of cancer cells [97]. Therefore, analysis of exosomal miRNAs from serum can be used for early disease detections or monitoring treatment responses and disease progressions of breast cancer. Plasma is also a common choice for exosomal miRNAs detection [51]. Although there may have some differences between plasma and serum in the level of free miRNAs, their levels of exosomal miRNAs are almost the same. In addition, Inubushi et al. [96] separated exosomal miRNAs from tear successfully and found that compared with healthy controls, the expression of breast-cancer-specific miR-200c and miR-21 was higher in tear exosomes of breast cancer patients, which indicates tear can be a potential source for breast cancer related exosomal miRNAs detection.

For the analysis of breast cancer-related exosomal miRNAs, there are mainly two strategies: separating exosomes firstly followed by miRNAs extraction and detection; testing the miRNAs in the serum directly without isolation. For the first strategy, exosomes are usually separated by ultracentrifugation, which is the traditional and most commonly used method for exosome separation [98]. In brief, cells and cell fragments are removed by centrifuging at 1000× *g* for 10 min followed by centrifuging at 10,000× *g* for 30 min to remove larger vesicles. After filtration using a 220 nm filter membrane, the filtrate is transferred into a centrifuge to precipitate and wash exosomes at 100,000~120,000× *g*. Other methods such as microfluidic-based strategies have also been reported for the separation of breast cancer-related exosomes [99,100]. miRNAs extraction is performed on the isolated exosomes by using nucleic acid extraction kits, some biochemical analysis can be carried out on the extracted miRNAs subsequently. For the second strategy, the development of biosensors and molecular beacons makes direct detection of miRNAs in complex body fluids possible, and these will be introduced in the detection approaches in detail.

### 3.2. Exosomal miRNAs Related to Breast Cancer Diagnosis

Multiple miRNAs have been identified for breast cancer diagnosis, even for distinguishing breast cancer subtypes [84,101]. For example, miR-423-5p [102], miR-18a-3p [99], miR-101, miR-372 [103], and eight miRNAs of miR-106a-363 cluster [104] which are associated with cancer proliferation, migration, and cell properties, can distinguish breast cancer patients with healthy ones. Other miRNAs, such as miR-373, are higher in triple-negative patients than that in luminal cancer patients or healthy controls; miR-223-3p [105], is higher in invasive ductal carcinoma patients than that in diagnosed preoperatively with ductal carcinoma in situ; and miR-93 [106], is also upregulated in ductal carcinoma in situ.

Single miRNA may have limitations in the breast cancer diagnosis included but not limited to low sensitivity and low specificity, resulting in low accuracy for breast cancer diagnosis. Combining multiple miRNAs together is a good solution. Jang et al. [87] chose four miRNAs (miR-373, miR-24, miR-206, and miR-1246) as biomarkers for breast cancer detection and they achieved the specificity of 96% and the sensitivity of 98% with an accuracy of 97%.

Despite distinguishing disease patients from healthy ones, exosomal miRNAs can also use to judge the therapeutic effects and prognosis of patients [107]. Bao et al. [108] identified three genomic instability-derived miRNAs (miR-421, miR-128-1, and miR-128-2), which can be used as minimally invasive biomarkers for poor prognosis. Sueta et al. [109] found three upregulated miRNAs (miR-124-3p, miR-340-5p, and miR-338-3p) and eight downregulated miRNAs (miR-29b-3p, miR-20b-5p, miR-17-5p, miR-130a-3p, miR-18a-5p, miR-195-5p, miR-486-5p, and miR-93-5p), which may be useful biomarkers for recurrences. Exosomal miRNAs associated with breast cancer diagnosis are shown in Table 2.

### 3.3. Detection Approaches

**Figure 2:**
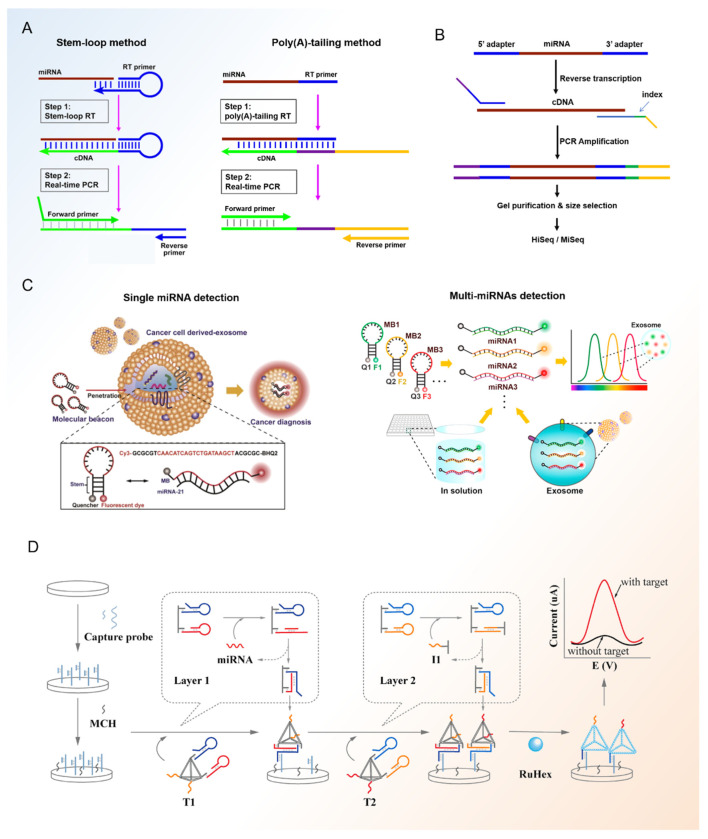
Detection approaches for breast cancer-related exosomal miRNA analysis. (**A**) RT-qPCR method for exosomal miRNA analysis, the length of miRNA was increased by stem-loop (left) or poly (**A**) tailing (right) method [112]. (**B**) Flow chart for miRNA sequencing. The length of miRNA was increased by an adapter at both 3′ and 5′ sides. (**C**) Molecular beacon for single (left) [110] and multiple (right) [113] exosomal miRNA analysis. (**D**) Schematic representation of the electrochemical platform for exosomal miRNA detection [114].

Exosomal miRNAs play an important role in the diagnosis of breast cancer. The great interest in these molecules has led to the significant development and continuous release of detection methods for basic and advanced exosomal miRNA diagnosis. In the following article, we outline several methods for breast cancer-related exosomal miRNA analysis (Figure 2).

#### 3.3.1. RT-qPCR

RT-qPCR (quantitative reverse transcription-polymerase chain reaction) is a technology combining real-time fluorescence quantification, reverse transcription (RT) of RNA and polymerase chain amplification (PCR) of cDNA. It has been used as a “gold” standard for nucleic acid assay [115]. The process of RT-qPCR contains two steps: using reverse transcriptase to synthesize cDNA from RNA; using DNA polymerase to amplify and synthesize target fragment with cDNA as template and the fluorescence signals in the reaction process are collected for real-time monitoring. Compared with RNAs, miRNAs are shorter with only 20–25 nucleotides. It is usually necessary to increase its length during the reverse transcription process by poly (A)-tailing [116] or stem-loop [117] method.

RT-qPCR has been widely used in the detection of breast cancer-related miRNAs. Li et al. [118] used RT-qPCR to screen candidate miRNAs for breast cancer detection. They profiled miRNA expression in plasma-derived exosome samples from 32 breast cancer patients and 32 normal controls and found miR-122-5p was significantly up-regulated in the plasma-derived exosome of breast cancer patients. Chen et al. [119] used 24 serum samples from clinical breast cancer and breast fibroma patients and found miR-18a-3p might have the potential to be a new biomarker to distinguish breast cancer from breast fibroma by using miRNA sequencing combing with RT-qPCR. In addition to screening the potential biomarkers, RT-qPCR can also help to explore the functions of exosomal miRNAs in the process of breast cancer. Zhao et al. [37] verified exosomal miRNA-205 might promote drug resistance and tumorigenesis in breast cancer with the help of RT-qPCR. The source of exosomes in this article was a human breast cancer cell line. Sueta et al. [109] compared miRNAs derived from exosome between breast cancer patients with recurrence (*n* = 16) and without recurrence (*n* = 16) by miRNA PCR array and identified four miRNAs (miR-340-5p, miR-17-5p, miR-130a-3p, and miR-93-5p) which were significantly associated with recurrence of breast cancer. In general, RT-qPCR is one of the major methods for exosome identification. It can quantify miRNAs accurately, but it can only detect miRNAs with known sequences.

#### 3.3.2. MiRNA Sequencing

MiRNA sequencing is another commonly used method for exosomal miRNAs detection, especially for the analysis of unknown miRNAs in samples. It can provide us with various information of miRNAs including length, sequence, structure, and content. Combing with GO (Gene Ontology) or KEGG (Kyoto Encyclopedia of Genes and Genomes) pathway database, we can also speculate the signal pathways associated with target miRNAs and explore some biological mechanisms [120].

In breast cancer, miRNA sequencing is often used to screen biomarkers for breast cancer diagnosis and treatment. Wu et al. [121] used miRNA sequencing to identify three healthy controls and 27 breast cancer patients and these cases were followed up for two years. They found 54 differentially expressed miRNAs that could distinguish triple-negative breast cancer patients with healthy controls and 3 miRNAs which could assess the risk of recurrence of breast cancer. Zhang et al. [122] isolated plasma-derived exosomes from seven post-chemotherapy patients and discovered miR-1-3p might be associated with anthracycline-induced liver injury during the chemotherapy for breast cancer patients with the help of miRNA sequencing. Despite their high price and cumbersome operation steps, miRNAs sequencing plays an irreplaceable role in the study of breast cancer exosomal miRNAs, especially in the search of disease mechanisms and new biomarkers for breast cancer diagnosis and subtypes distinguishment. With the abundance of the sequencing library, breast cancer-related exosomal miRNAs database can be established and new sequencing samples can be classified with the help of artificial intelligence [123].

#### 3.3.3. Molecular Beacons

Both RT-qRCR and miRNA sequencing methods need pre-separation of exosomes before the test which may cause loss of target analytes. Moreover, they are laborious and time-consuming which makes them unsuitable for high-throughput exosomal miRNA detection for diagnosis in clinical. Under this premise, more and more attention has been paid to PCR-free diagnosis methods. A molecular beacon is a dual-labeled oligonucleotide hairpin probe with a fluorophore and a quencher at each end [124,125]. This stem and loop structure has low background fluorescence and high specificity, making molecular beacon a suitable probe for the imaging of RNAs in cells directly [126]. Excess unreacted molecular beacons do not need to be removed from the reaction system since they have self-quenching ability. In order to confirm the specificity of the molecular beacon, it is necessary to design and screen the target sequences.

Nowadays, miRNAs have been quantified successfully by a molecular beacon with high specificity [127] in breast cancer. Lee et al. [110] detected miR-21 in the exosomes from breast cancer cells successfully by using molecular beacons. Streptolysin O was used to improve the permeability of exosome membranes, thus enhancing the transmission of molecular beacons into exosomes and increasing the signal of target miRNA. Furthermore, they investigated a simultaneous and multiplexed detection method of breast cancer-related exosomal miRNAs in their following work [113]. They chose miR-21, miR-375, and miR-27a as the target miRNAs. In order to realize simultaneous detection, the fluorescent dye of different molecular beacons has different excitation wavelengths such as Cy5 and FAM. Using this method, they detected multiple miRNAs in breast cancer cell line derived-exosomes successfully within 1 h. Due to the low abundance of miRNAs in exosomes, some strategies which can increase the concentration of miRNAs including rolling circle amplification have also been used in the molecular beacon detection process of breast cancer related exosomal miRNAs [128].

#### 3.3.4. Other miRNA Biosensors

Besides molecular beacons, other biosensor strategies have also been used in the detection of breast cancer -exosomal miRNAs. Zhang et al. [114] proposed an electrochemical biosensor for exosomal miRNA analysis using multifunctional DNA tetrahedrons assisted catalytic hairpin assembly (MDTs-CHA). The electrochemical platform can measure exosomal miRNAs of breast cancer quantitatively in 30 min with good specificity. In addition, by profiling four breast cancer-related exosomal miRNAs (miR-375, miR-21, miR-1246, and miR-221), the platform showed high sensitivity (90.5%) and efficiency (AUC:0.989) for the diagnosis of serum from 9 healthy donors and 21 breast cancer patients. Wang et al. [129] designed an all-in-one biosensor based on a DNA three-way junction that can realize the detection of three miRNAs (miR-21, miR-375, and miR-27a) simultaneously. Based on the integration of multiple recognition sequences, the biosensor can ensure that three different sensing probes are transmitted into the exosomes equivalently, thus, reducing signal interference and improving the accuracy of multiple detections of exosomal miRNAs. Using this biosensor, the author differentiated the serum of three breast cancer patients from two healthy controls effectively. In order to improve the sensitivity, some ultrasensitive detection methods such as surface-enhanced Raman scattering [130] combing with nucleic acid probes have also been used in the detection of breast cancer-related exosomal miRNAs. Overall, biosensors including molecular beacons have benefits in the high-throughput diagnosis of breast cancer in clinical, but efforts are still needed in their design, sensitivity, and standardization.

## 4. Conclusions

There is growing evidence to support the emerging role of exosomal miRNAs in tumorigenesis, proliferation, metastasis, and drug resistance. The identification of breast cancer-specific exosomal miRNAs and their potential mechanism will help early diagnosis of disease, determine the sensitivity to therapeutic drugs, and formulate appropriate treatment strategies. In addition, the breast cancer process can be controlled by regulating specific miRNAs through exosomes. For example, Samaneh et al. [131] used mesenchymal stem cell-derived exosomes to deliver miR-381-3p to inhibit triple-negative breast cancer aggressiveness; Ohno et al. [132] injected exosomal let-7a to breast cancer tissue for anti-tumor; and Kim et al. [133] used let7c-5p for breast cancer therapy. Exosomal miRNAs therapy will be a new strategy for breast cancer treatment. Besides, some techniques, such as molecular beacons, next-generation sequencing, microarrays, and miRNA enzyme immunoassay have made the detection of breast cancer based on miRNAs possible.

However, there are still have some difficulties in exosomal miRNAs applications in clinical. Establishing standards is one of the major limitations in exosomal miRNAs-based breast cancer diagnosis. Most of the existing methods are based on small numbers of samples and miRNAs are detected using different methods. Although in these articles, breast cancer and health groups can be well-differentiated, there is no clear numerical range to identify breast cancer. It is very necessary to test a large number of samples and establish standard test methods. Specificity is another limitation for exosomal miRNAs application in breast cancer. Many miRNAs reported now are not breast cancer-specific, such as miR-21. Combining multiple means and detecting multiple miRNAs at one time are expected to improve the detection accuracy. In conclusion, exosomal miRNAs play an important role in breast cancer progressions and may further be considered as an excellent biomarker for the prevention, early diagnosis, and treatment of breast cancer in the near future.

## Figures and Tables

**Table 1:** Exosomal miRNAs involved in breast cancer progression.

Exosomal miRNA	Target Gene/Signal Pathway	Function	Ref.
miR-205	E2F1	Affect breast cancer cells proliferation	[37]
miR-9	E-cadherin	Regulate cancer-related fibroblast induce	[39]
miR-210	Ephrin A3 and PTP1B	Promote angiogenesis	[40]
miR-181d-5p	CDX2/HOXA5	Promote EMT	[79]
miR-21	PDCD4	Facilitate osteoclastogenesis	[49]
miR-363-5p	PDGFB	Modulate platelet-derived growth factor	[51]
miR-7641	HIF-1 alpha	Promote the stemness of breast cancer cells	[53]
miR-200	ZEB2 and SEC23A	Promote metastatic capability	[80]
miR-155	DUSP14	Enhance metastasis	[54]
miR-1226-3p	AQP5	Inhibit migration	[55]
miR-130a-3p	RAB5B	Inhibit migration and invasion	[56]
miR-183-5p	PPP2CA	Enhance IL-1β, IL-6 and TNF α expressions	[60]
miR-27a-3p	MAGI2/PTEN/PI3K	Increase PD-L1 expression	[61]
miR-181a and miR-9	SOCS3 and PIAS3	Promote the expansion and infiltration of immature eMDSCs	[62]
miR-127, miR-197, miR-222 and miR-223	CXCL12	Contribute to breast cancer cell quiescence	[50]
miR-770	STMN1	Suppress chemo-resistance and metastasis	[81]
miR-100, miR-222 and miR-30a	PTEN	Resist to adriamycin and docetaxel	[67]
miR-9-5p	ADIPOQ	Resist to tamoxifen	[75]
miR-1246	CCNG2	Promote cell proliferation, invasion and drug resistance	[82]
miR-567	ATG5	Reverse trastuzumab resistance	[83]

**Table 2:** Exosomal miRNAs involved in breast cancer diagnosis.

Exosomal miRNA	Application	Expression	Ref.
miR-423-5p, miR-21, miR-1246	Distinguish breast cancer patients from healthy ones	Upregulated	[102,110,111]
miR-18a-3p	Distinguish breast cancer from breast fibroma	Upregulated	[99]
miR-373	Distinguish triple-negative patients from luminal cancer patients and healthy controls	Upregulated	[103]
miR-101, miR-372	Distinguish breast cancer from benign tumors	Upregulated	[103]
miR-106a-363 cluster	Breast cancer diagnosis	Upregulated	[104]
miR-223-3p	Distinguish invasive ductal carcinoma from ductal carcinoma in situ	Upregulated	[105]
miR-16	Distinguish breast cancer and ductal carcinoma in situ from healthy women	Upregulated	[106]
miR-30b	Predict recurrence	Downregulated	[106]
miR-93	Ductal carcinoma in situ diagnosis	Upregulated	[106]
miR-373, miR-24, miR-206 and miR-1246	Breast cancer detection	Upregulated	[87]
miR-421, miR-128-1 and miR-128-2	Predict risk and unfavorable prognosis	Upregulated	[108]
miR-340-5p	Predict recurrence	Upregulated	[109]
miR-17-5p, miR-130a-3p, and miR-93-5p	Predict recurrence	Downregulated	[109]
miR-155, miR-301	Predict pathological complete response	Before therapy: upregulated After therapy: downregulated	[107]
